# Prognosis in Various Forms of Paralysis

**Published:** 1884-02-20

**Authors:** Philip Zenner


### PROGNOSIS IN VARIOUS FORMS OF PARALYSIS.

By PHILIP ZENNER, A.M., M.D.

To the laity the word paralysis seems always to have the same grave significance. They do not know that, depending on the underlying condition, this symptom may be of but trifling consequence, as well as a source of lasting disability, or a precursor of an early end. Unfortunately, even physicians sometimes associate with this word an unvarying meaning, one of evil augury, without attempting to determine the condition upon which the symptom depends.

I wish, in this paper, to speak briefly of some of the more prominent forms of paralysis with especial reference to their prognosis, and though the latter cannot be properly determined without previous knowledge of the exact lesion or disease in each instance, yet a general consideration of the gravity of the various forms, and of means which often assist us to properly determine the prognosis in the individual case, will, I trust, be both instructive and interesting.

We will first consider the most common form of paralysis of central origin—hemiplegia.

In the great majority of cases this is due either to embolism of the middle cerebral artery or its branches, or to hemorrhage in the vicinity of the corpus striatum. There is paralysis of the limbs and face on the side opposite to the seat of lesion. If the patient recovers from the apoplectic attack, which usually ushers in the paralysis, and his life is no longer in immediate danger, the important question arises, will he be permanently disabled by the paralysis, or will the latter entirely or largely disappear? The answer to this question will mainly depend on the seat of the lesion—on what part of the brain has been injured. When only the large basal ganglia have been injured, the paralysis usually disappears after a lapse of time, but when the internal capsule has been destroyed the paralysis is permanent. Whether the latter has, or has not occurred, is very soon revealed by the symptoms. If the internal capsule has been destroyed, there is subsequent degeneration of the pyramidal strands in the spinal cord, attended by such symptoms as rigidity of the paralyzed limbs, and exaggerated tendon reflexes. The rigidity of the paralyzed muscles is first observed after some time, usually months, after the original injury; but the exaggerated tendon reflexes (the foot clonus, exaggerated patellæ, tendon reflex, etc.) may already be detected within a few days or weeks. They, therefore, at a very early day will assist us to a correct prognosis.

So, when we are called to a case of hemiplegia, and wish to determine whether the paralysis will be temporary or permanent, we should examine the tendon reflexes. If we find them to be exaggerated, we may already surmise that permanent injury has been done; and if, after a few weeks or months, rigidity of the paralyzed muscles is also observed, that surmise becomes positive knowledge.

As a general rule the prognosis of spinal paralysis is less favorable than that of cerebral origin. But among the various forms of spinal paralysis, some that present the most marked motor symptoms have, in reality, the most favorable prognosis. Thus, we often find with Pott's disease of the spine a high degree of paraplegia which may, later, partly or entirely disappear.

The paralysis is not produced, as was long supposed, by direct pressure on the cord from the angular curvature, but is due to secondary changes in the meninges or substance of the cord. The symptoms presented are those usually attributed to sclerosis of the lateral columns, paraplegia, rigidity of the limbs, often convulsive movements in the paralyzed parts, and greatly exaggerated tendon reflexes. In these cases, even though the paralysis be of some years' standing, improvement may be expected with much confidence after the acute changes in the bone have subsided.

In the disease termed spastic spinal paralysis, supposed to be due to primary sclerosis of the lateral columns of the cord, and presenting symptoms exactly similar to those just described, the prognosis is also more favorable than the marked symptoms would suggest; more favorable, in fact, than that of most of the organic diseases of the cord.

Let us next consider peripheral paralyses with special reference to the means of determining their prognosis. There are very many forms of peripheral paralysis due to traumatic or other causes, and also all degrees of intensity, sometimes being only slight and transient, at other times complete and lasting.

Let us take for illustration two of the most common forms, facial paralysis and paralysis of the muscles supplied by the radial nerve. One awakes in the morning and finds that the face is drawn to one side, that the eyelids cannot be closed—there is facial paralysis. There is no apparent cause. It is termed rheumatic paralysis. Probably the sheath of the nerve somewhere within the temporal bone is swollen and the nerve thus compressed. Again, we arise in the morning and find the muscles supplied by the radial nerve paralyzed. The patient had probably been lying upon that arm and thus compressed the nerve trunk. We find what is termed wrist-drop. The appearance is like that of lead paralysis, but it has come on suddenly and is limited to one side.

In either of these cases, especially the latter, the prognosis is generally favorable; the result, full recovery. But the paralysis may be of very short duration; it may be of long duration, and sometimes it is permanent.

Have we any means by which we can determine, then, the prognosis in each case? Yes, in the electrical reaction of the nerves and muscles.

Normally the muscles contract when electricity is applied to them directly, or to the nerves supplying them. But the electrical reaction may be quite different as a result of pathological changes. The most prominent changes are those termed the reaction of degeneration. Here the muscles do not contract when electricity is applied to the nerve supplying them; they do not contract when the interrupted or faradic current is applied directly to the muscles, but do contract, in a different manner from the normal, when the constant, or galvanic current is applied directly to the muscles.

Now, let us revert to our case of facial paralysis. If no change takes place in the electrical reaction, but the latter remains as it is normally, the paralysis will be of short duration, and a full recovery will ensue. But if the reaction of degeneration is soon observed, paralysis will be of long standing; recovery will be slow, perhaps incomplete; or the paralysis may be lasting.

In some cases the reaction of degeneration becomes only partly developed. Here the prognosis is more favorable, though less so than where no changes occur in the electrical reaction.

The same test will assist us in the prognosis of one case of paralysis of muscles supplied by the radial nerve as well as in all other cases of peripheral paralysis.

I will next mention a class of cases in which the prognosis is far more favorable than the clinical picture presented would, at first sight, lead us to suppose; that is, the paralysis following infectious diseases. We most frequently find these after attacks of diphtheria. The paralysis of the soft palate, muscles of the eye, etc., are known to you all. Their prognosis is nearly always favorable. But these are not the only paralyses caused by diphtheritic poisoning. There is, less frequently, paralysis of spinal origin, paraplegia, ataxia, etc., which may be very marked, even alarming in their appearance, but whose prognosis is, nevertheless, as favorable as that of the more common forms of diphtheritic origin.

The prognosis of this class of cases illustrates the fact that in forming a proper judgment of the gravity of a case, it is often necessary to know not only the seat and extent of local damage, but also something of the character of the pathological process, to know the course of the disease. Thus, we have learned of diphtheritic paralysis that, while apparently very serious, they usually terminate favorably. In some other diseases we will find that, while the paralytic manifestations, especially in the beginning, may be very mild, we must expect the most unfavorable termination.

In cases of multiple sclerosis of the brain and spinal cord, though the symptoms presented may be slight, we know that the tendency of the disease is to extend, and, though very slow in its progress and variable in its manifestations, it will not terminate in recovery. The same may be said, with some modifications, of locomotor ataxia.

These diseases, though tending to extend, do not, as a rule, immediately threaten life, while some others lead directly to a fatal termination. Thus, amyotrophic lateral sclerosis, attended by paralysis and wasting of muscles, causes death, through involving the medulla oblongata. within a few years of its inception.

Progressive muscular atrophy terminates in the same manner, at a much later date. Some acute spinal paralysis, notably Lantry's paralysis, may terminate fatally within a few weeks, while others, not unlike the latter in general appearance, as some cases of subacute anterior poliomyelitis may terminate in full recovery.

In all these cases, in order to arrive at a proper prognosis, we must first make a correct diagnosis and know the course of the disease.—*Louisville Medical News.*

